# Molecular Identity and Location Influence Purkinje Cell Vulnerability in Autosomal-Recessive Spastic Ataxia of Charlevoix-Saguenay Mice

**DOI:** 10.3389/fncel.2021.707857

**Published:** 2021-12-14

**Authors:** Brenda Toscano Márquez, Anna A. Cook, Max Rice, Alexia Smileski, Kristen Vieira-Lomasney, François Charron, R. Anne McKinney, Alanna J. Watt

**Affiliations:** ^1^Department of Biology, McGill University, Montreal, QC, Canada; ^2^Department of Pharmacology and Therapeutics, McGill University, Montreal, QC, Canada

**Keywords:** ataxia, mouse models, patterning, cerebellum, Purkinje cell, zebrin/Aldolase C

## Abstract

Patterned cell death is a common feature of many neurodegenerative diseases. In patients with autosomal-recessive spastic ataxia of Charlevoix-Saguenay (ARSACS) and mouse models of ARSACS, it has been observed that Purkinje cells in anterior cerebellar vermis are vulnerable to degeneration while those in posterior vermis are resilient. Purkinje cells are known to express certain molecules in a highly stereotyped, patterned manner across the cerebellum. One patterned molecule is zebrin, which is expressed in distinctive stripes across the cerebellar cortex. The different zones delineated by the expression pattern of zebrin and other patterned molecules have been implicated in the patterning of Purkinje cell death, raising the question of whether they contribute to cell death in ARSACS. We found that zebrin patterning appears normal prior to disease onset in *Sacs^–/–^* mice, suggesting that zebrin-positive and -negative Purkinje cell zones develop normally. We next observed that zebrin-negative Purkinje cells in anterior lobule III were preferentially susceptible to cell death, while anterior zebrin-positive cells and posterior zebrin-negative and -positive cells remained resilient even at late disease stages. The patterning of Purkinje cell innervation to the target neurons in the cerebellar nuclei (CN) showed a similar pattern of loss: neurons in the anterior CN, where inputs are predominantly zebrin-negative, displayed a loss of Purkinje cell innervation. In contrast, neurons in the posterior CN, which is innervated by both zebrin-negative and -positive puncta, had normal innervation. These results suggest that the location and the molecular identity of Purkinje cells determine their susceptibility to cell death in ARSACS.

## Introduction

Autosomal-recessive spastic ataxia of Charlevoix-Saguenay (ARSACS) is a progressive neurodegenerative disorder (Bouchard et al., 1978) caused most often by a deletion mutation in the *SACS* gene encoding the large (521 kD) protein sacsin (Engert et al., 2000) that functions as a chaperone (Parfitt et al., 2009) and regulates the cytoskeleton (Duncan et al., 2017; Gentil et al., 2019). Mouse models of ARSACS have been developed that recapitulate key disease phenotypes, such as ataxia and progressive loss of cerebellar Purkinje cells (Girard et al., 2012; Lariviere et al., 2015, 2019). Remarkably, Purkinje cell death is patterned in human patients (Bouchard et al., 1998) and mouse models (Lariviere et al., 2015, 2019): cell death is more prominent in Purkinje cells in the anterior vermis while Purkinje cells in the posterior vermis are largely spared. The factors that cause anterior Purkinje cells to be vulnerable to changes in cellular function caused by the absence of sacsin have yet to be determined.

Patterning is a striking feature of the molecular identity of Purkinje cells. Purkinje cells express aldolase C, also known as zebrin II (zebrin), in a distinctive, striped pattern across the cerebellum that is evolutionarily conserved (Brochu et al., 1990). More recently, other molecules have been shown to be expressed in a patterned distribution across the cerebellum (Wadiche and Jahr, 2005; Apps and Hawkes, 2009; Marzban and Hawkes, 2011). How the molecular identity of neurons affects their function is not completely understood. Remarkably, however, recent evidence suggests that the molecular identity of Purkinje cells and their location within the cerebellum confer differences in both synaptic (Wadiche and Jahr, 2005; Hawkes, 2014) and firing properties (Zhou et al., 2014; Perkins et al., 2018).

The observation that many diseases show specific deficits related to molecular patterning underscores the urgency of understanding cerebellar patterning in more depth (Sarna and Hawkes, 2003). For instance, some diseases display Purkinje cell death predominantly in specific regions of the cerebellum, such as regions that are comprised of largely zebrin-negative or zebrin-positive cells (Sarna and Hawkes, 2003). One intriguing hypothesis is that diseases that share common vulnerabilities in Purkinje cells of a particular molecular identity may share pathophysiology that could be targeted by the same therapeutic interventions. It is therefore important to identify whether a neurodegenerative disease displays patterning in its cell loss and whether that patterning is associated with a specific molecular identity. We thus wanted to determine whether the patterned cell death observed in ARSACS arose from changes in specific populations of Purkinje cells that express specific molecular markers.

To shed light on whether Purkinje cells of a particular molecular identity are vulnerable to cell death, we explored whether zebrin expression is altered in a mouse model of ARSACS. We found that there were no changes in the zebrin patterning in *Sacs^–/–^* mice prior to Purkinje cell death, suggesting that the developmental sequences of patterning (Larouche and Hawkes, 2006) are unaltered in ARSACS. Interestingly, however, we found that Purkinje cell death occurs predominantly in zebrin-negative cells in the anterior vermis, even at later disease stages when Purkinje cell death is more widespread. Finally, we show that the deficit that we had previously observed in Purkinje cell synaptic innervation in the cerebellar nuclei (CN) (Ady et al., 2018) is also shaped by zebrin identity and anterior-posterior divisions. We observed that the reduction of Purkinje cell inputs onto CN neurons is only observed for zebrin-negative Purkinje cell terminals made onto cells in the anterior CN. In contrast, Purkinje cell innervation to the posterior CN, which is comprised of both zebrin-negative and -positive puncta, appears unaffected. These findings support the hypothesis that zebrin-negative Purkinje cells in the anterior vermis are uniquely susceptible to Purkinje cell death in ARSACS.

## Materials and Methods

### Animals

*Sacs^–/–^* mice carrying a deletion of the *Sacs* gene were used as previously described (Girard et al., 2012; Lariviere et al., 2015; Ady et al., 2018). Heterozygous *Sacs*^+/–^ mice were bred to obtain litter-matched *Sacs*^–/–^ and wildtype (WT) mice. Roughly equal numbers of male and female mice were used in all experiments. Sex differences were tested for but not observed. Experiments were performed at postnatal day 40 (P40; *N* = 4 for WT and *N* = 4 for *Sacs^–/–^* mice), P150 (*N* = 7 for WT and *N* = 8 for *Sacs^–/–^* mice), P270 (*N* = 4 for WT and *N* = 4 for *Sacs^–/–^* mice), and P365 (*N* = 4 for WT and *N* = 4 for *Sacs^–/–^* mice). Breeding and animal procedures were approved by the McGill University Animal Care Committee and were in accordance with the rules and regulations established by the Canadian Council on Animal Care.

### Tissue Preparation and Immunohistochemistry

Brain tissue from WT and *Sacs^–/–^* mice was prepared as previously described (Ady et al., 2018). In brief, mice were deeply anesthetized with 2,2,2-tribromoethanol (Avertin) via intraperitoneal injection, and tissue fixation was carried out via intracardiac perfusion. An initial flush was performed with phosphate-buffered saline (PBS, 0.1M, pH 7.4) and 5.6 μg/ml heparin salt. This was followed by perfusion with 40 ml of 4% paraformaldehyde (PFA) in phosphate buffer (PB, pH 7.4), which was also used for post-fixation storage of the extracted brains for a further 3 days at 4°C on a shaker at 70 RPM. If further storage was required, brains were stored in PBS with 0.5% sodium azide until slicing.

The cerebellum was sliced coronally using a Vibratome 3000 sectioning system (Concord, ON, Canada) to produce 100 μm thick slices. Immunohistochemistry was carried out on free-floating slices and two sections per location were used for each animal. Briefly, slices were incubated for half an hour in blocking solution (containing 1 × PBS, pH 7.4, 0.4% Triton X, 5% bovine serum albumin (BSA), and 0.05% Sodium Azide) followed by a 3-day incubation with the primary antibodies in blocking solution and 90 minutes for secondary antibodies in blocking solution. Following the final incubation, slices were washed in PBS and mounted using ProLong Gold Antifade mounting medium (ThermoFisher Scientific, Waltham, MA, United States), protected from light, and kept at 4°C. All chemicals were purchased from Millipore Sigma (Oakville, Canada) unless otherwise indicated.

*Lobule immunohistochemistry:* To label zebrin-positive cells, we used goat Aldolase C antibody (1:300; Catalogue number SCl-12065; Santa Cruz Biotechnology, Dallas, TX, United States) in combination with Alexa-488 conjugated anti-goat antibody (A11055; Life Technologies, Burlington, ON, Canada). In addition, we used rabbit anti-calbindin antibody (1:500, CB-38a; Swant, Marly, Switzerland) as a Purkinje cell marker in combination with Alexa-594 conjugated anti-rabbit secondary antibody (R37119; Life Technologies). All secondary antibodies were used at 1:1,000 dilution. In a separate set of experiments to validate the use of calbindin staining to determine Purkinje cell numbers in Supplementary Figure 1, we performed immunohistochemistry as previously described for calbindin, followed by a counterstaining step using NeuroTrace 435/455 blue fluorescent Nissl stain (1:80; 1-hour incubation; N21479; Life Technologies).

*Cerebellar nuclei immunohistochemistry:* To label Purkinje cell terminal puncta in the CN, sagittal cerebellar slices were prepared and stained with rabbit anti-calbindin antibody (1:500, CB-38a, Swant) in combination with an Alexa-594 conjugated anti-rabbit secondary antibody (711-585-152, Jackson Immunoresearch, West Grove, PA, United States). To label zebrin-positive Purkinje cell puncta, mouse aldolase-C antibody (1:500, ab190368, Abcam, Toronto, Canada) was used in combination with Alexa-488 anti-mouse secondary antibody (715-545-150, Jackson Immunoresearch). CN neurons were labeled with guinea pig anti-NeuN antibody (1:500, abN90, Millipore, Burlington, MA, United States) in combination with DyLight 405 anti-guinea pig secondary antibody (1:500, 106475003, Jackson Immunoresearch). In separate experiments on additional slices from the same experimental animals, guinea pig vesicular GABA transporter (VGAT) antibody (1:200, 131 004, Synaptic Systems, Gottingen, Germany) with Alexa-488 anti-guinea pig secondary antibody (1:1000, 706-545-148, Jackson Immunoresearch) was used in conjunction with rabbit anti-calbindin antibody (Swant, as before), to label GABAergic terminals in the CN, shown in Supplementary Figure 2. For each animal, eight sagittal slices were collected at spaced intervals to ensure sampling across the fastigial and interposed CN. We used four WT and three *Sacs^–/–^* P90 mice. Number of cells: WT anterior = 28 cells, WT posterior = 26 cells, *Sacs^–/–^* mice anterior = 25 cells, *Sacs^–/–^* mice posterior = 46 cells.

### Image Acquisition and Analysis

*Zebrin stripe imaging*: Images were acquired using an Axio Observer Z1 fluorescent microscope equipped with Zen blue software (Zeiss, Oberkochen, Germany) at a 20× magnification and tiled together for a whole lobule visualization. Gain and contrast were kept constant for all conditions during the imaging process. To compare across conditions, we used anatomical markers in the cerebellum to ensure that we were comparing data at the same brain location. We selected the cerebellar slices using images from the coronal view of the interactive Allen Mouse Brain atlas viewer^1^ (Allen Institute of Brain Science, mouse coronal Atlas, 2011). For the analysis of the anterior lobules, we selected sections that matched images between 115 and 117; and for the posterior lobules, we selected sections that matched images between 127 and 130. We used two sections per lobule per animal. Mean fluorescence intensity and band thickness were measured using ImageJ 1.51F software. Briefly, the mean intensity was measured by tracing a box that contained both soma and dendrites of each zebrin-positive band. A similar box (size and position) was used to measure the zebrin-negative bands. To measure the thickness of the bands, a line containing only the zebrin-positive label was traced parallel to the length of the lobule, midway of the length of the dendritic tree. Complementary measurements were done in the zebrin-negative bands (refer to Supplementary Figure 3 for illustration of analysis).

*Purkinje cell counts:* Images were acquired at a 20× magnification. Images were taken in one focal plane where the label of both primary antibodies was seen. In a separate set of experiments, we confirmed using a Nissl stain that calbindin gave us a good measurement of total Purkinje cell number (Supplementary Figures 1, 3), at an age when Purkinje cell death was observed. Thus, calbindin labeling was used to count total Purkinje cell numbers, while zebrin labeling was used to discern zebrin-positive (that were co-labeled for calbindin and zebrin) cells from zebrin-negative cells (that were labeled only for calbindin), even when Purkinje cell death was evident. We measured Purkinje cell density by measuring the number of Purkinje cells as a function of the length of the Purkinje cell layer, reporting numbers per 100 μm. The same measurement was used for zebrin-positive and zebrin-negative cells. Measurements were performed on sections that were identified using coordinates from the Allen Mouse Brain Atlas as described above. Briefly, anterior lobule III and possibly the boundary of lobule IV were identified between positions 115 and 117, and posterior lobules (lobules VIII and IX) were identified between positions 127 and 130. Total Purkinje cell length per condition was similar and is reported in Table 1. Representative images of the immunostaining are shown in pseudo-color.

**TABLE 1:** Animal and section number per condition.

Age	Anterior Lobule				Posterior Lobule			
	WT		*Sacs* ^–/–^		WT		*Sacs* ^–/–^	
	*N* animals (sections)	Total length of Purkinje cell layer analyzed (mm)	*N* animals (sections)	Total length of Purkinje cell layer analyzed (mm)	*N* animals (sections)	Total length of Purkinje cell layer analyzed (mm)	*N* animals (sections)	Total length of Purkinje cell layer analyzed (mm)
P40	4 (8)	37.19	4 (8)	38.33	4 (8)	35.54	4 (8)	37.34
P150	7 (14)	73.94	8 (16)	73.39	4 (8)	34.01	4 (8)	37.14
P270	4 (8)	37.48	4 (8)	37.99	4 (8)	38.43	4 (8)	37.94
P365	4 (8)	34.10	4 (8)	34.61	4 (8)	34.96	4 (8)	35.67

*Animal number and the number of sections used for each condition are indicated for both genotypes in anterior and posterior lobules. Total Purkinje cell layer length (in mm) used in the analysis is indicated for each comparison. For each animal and lobule (anterior, posterior), two slices were selected for the analysis and one image was taken per slice.*

*Cerebellar nuclei imaging:* Images were acquired using an LSM800 confocal microscope (Zeiss) at 1024 × 1024 resolution with a 63× objective and consistent imaging conditions and settings throughout. Images stacks of CN cells were obtained, and the cross-section of each cell at the widest point was chosen for analysis. The number of Purkinje cell synapses onto each CN cell was quantified by counting the number of calbindin-positive puncta surrounding large CN cells (>15 μm diameter). Puncta were only included in counts if they were immediately adjacent to the NeuN-positive CN cells. Puncta that were not touching the CN cell (located more than 0.5 μm away from the NeuN-positive area) were determined to not be forming a synapse with that CN cell and were not included in counts (refer to Supplementary Figure 2 for examples). Zebrin-positive Purkinje cell synapses were identified by counting the number of the previously identified calbindin-positive puncta that were also zebrin-positive (Supplementary Figure 2). The number of zebrin-negative Purkinje cell synapses was then determined by subtracting the number of zebrin-positive puncta from the total number of calbindin-positive puncta. The data were then collated to show the number of zebrin-positive or zebrin-negative Purkinje cell puncta per large CN cell. All imaging was acquired and analyzed blind to condition, and images are presented in pseudo-color.

### Statistics

Comparisons were made using paired or unpaired Student’s *t*-tests for normally distributed data or Mann Whitney *U* tests for when data were not normally distributed using JMP 12 (SAS, Cary, NC, United States) software. Data are represented by box and whisker plots, showing the median (horizontal line within boxes), second and third quartiles (rectangles) ± 1 SD (whiskers), or by an average ± SEM.

## Results

### Patterning of Purkinje Cell Death in Autosomal-Recessive Spastic Ataxia of Charlevoix-Saguenay Mouse Model

The anterior-posterior differences in Purkinje cell firing properties have been associated with the expression profile of zebrin (Larouche and Hawkes, 2006): zebrin-positive cells are enriched in posterior lobules and fire at a lower frequency than zebrin-negative cells, which are enriched in anterior lobules (Xiao et al., 2014; Zhou et al., 2014). Since abnormal zebrin expression has been observed in rodent models of other forms of ataxia (Sawada et al., 2009; Sarna and Hawkes, 2011; Bailey et al., 2014; White et al., 2021), the changes in anterior-lobule firing that we previously reported (Ady et al., 2018) may likewise arise from abnormal expression of zebrin in the cerebellum in *Sacs^–/–^* mice. Conversely, intact neurotransmission from Purkinje cells to their downstream targets is required for proper zone formation in the cerebellum (White et al., 2014), and given that we have previously shown that Purkinje cells in *Sacs^–/–^* mice both fire at reduced frequencies and have deficits in their innervation of the CN (Ady et al., 2018), we wondered whether changes in Purkinje cell properties could themselves lead to disrupted zebrin patterning.

**FIGURE 1:**
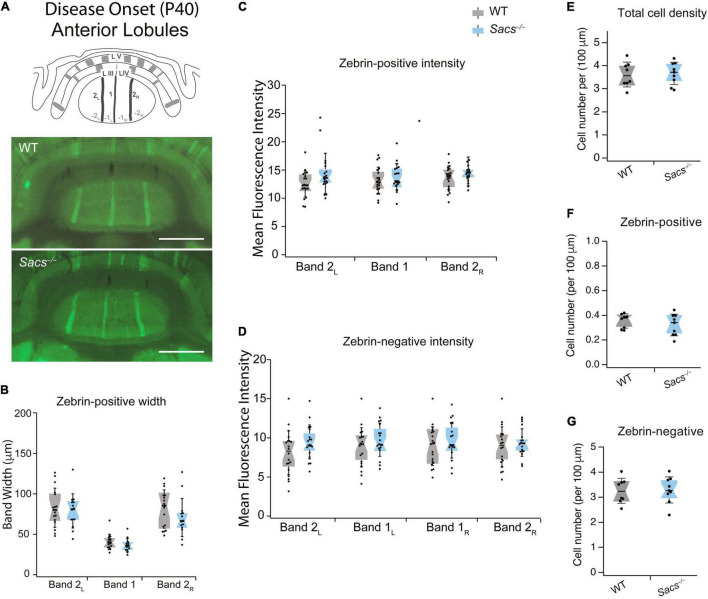
Anterior vermis zebrin expression is unaltered in disease-onset (P40) *Sacs^–/–^*mice. **(A)** Schematic (top) illustrates a coronal view of anterior lobules (L) and zebrin bands. Measurements were taken from anterior lobule III/IV at P40. Representative images show anterior zebrin expression (green) in WT (middle) and *Sacs^–/–^* mice (bottom). **(B)** The widths of the zebrin-positive bands in the anterior lobule were not significantly different in *Sacs^–/–^* cerebellum (blue) compared to WT (gray). **(C,D)** Similarly, there were no significant differences observed between the mean fluorescence intensity (arbitrary fluorescence units, a.u.) of **(C)** zebrin-positive bands **(D)** or zebrin-negative bands in WT (gray) and *Sacs^–/–^* (blue) cerebellar vermis. **(E–G)** No significant reduction was observed in the total density of Purkinje cells in anterior lobules [**(E)** shown as the number of cells per μm]. We also observed no differences in zebrin-positive **(F)** and zebrin-negative **(G)** cell densities. No significant differences were observed for any comparisons. *N* = 4 for both WT and *Sacs^–/–^* mice, two sections per animal; see Table 2 for *P*-values; Student’s *t*-test, *P* > 0.05 when no comparison is shown. Scale bar, 1 mm. WT, wildtype.

Anterior lobules (III and IV) are comprised of mostly zebrin-negative Purkinje cells, with only three thin, sagittally oriented zebrin-positive stripes in the vermis. We rationalized that if changes we observed in firing rates were due to changes in zebrin expression, they might arise either from the expansion of the width of zebrin stripes or from aberrant ectopic zebrin expression in regions that are normally zebrin-negative, which would be associated with lower Purkinje cell firing rates. We used immunohistochemistry in coronal slices from WT and *Sacs^–/–^* mice at disease onset (P40-50) to examine zebrin expression throughout anterior lobules III/IV (Figure 1A and Supplementary Figure 3). We found no significant differences in the width of zebrin-positive stripes when comparing WT and *Sacs^–/–^* cerebellum (*N* = 4 mice for WT and *Sacs^–/–^*, 2 sections/brain analyzed; Figure 1B, see Table 2 for individual *P*-values for each band). If developmental changes in zebrin patterning occurred in *Sacs^–/–^* mice, the intensity of zebrin staining in both zebrin-negative and -positive bands might be altered, since changes in molecular compartmentalization have been reported in other mice (Miyazaki et al., 2012; Bailey et al., 2014). We determined the intensity of zebrin staining in both zebrin-positive and zebrin-negative stripes in anterior lobules and found no significant differences in *Sacs^–/–^* mice compared to WT (Student’s *t*-test for all comparisons other than posterior bands 2_L_, 1_R_, and 2_R_ when Mann Whitney *U* tests were performed, not significantly different; zebrin-positive bands: band 2_L_: *P* = 0.217, band 1: *P* = 0.075, band 2_R_: *P* = 0.109; zebrin-negative bands: band 2_L_: *P* = 0.10, band 1_L_: *P* = 0.525, band 1_R_: *P* = 0.675, band 2_R_: *P* = 0.706; Figures 1C,D). This suggests that the reduction in Purkinje cell firing rates that we observed in anterior lobules at P40 (Ady et al., 2018) does not arise from abnormal expression of zebrin in these cells or from changes in the developmental patterning or expression of zebrin. Previously, it has been reported that Purkinje cell death is not observed at P30 but is evident at P90 (Lariviere et al., 2015). To determine whether cell death is observed at P40, we determined the density of Purkinje cell bodies and found that no differences were observed in *Sacs^–/–^* mice compared to WT (Student’s *t*-test, *P* = 0.99; Figure 1E). Similarly, there were no detectable differences in either zebrin-positive (Student’s *t*-test, *P* = 0.37; Figure 1F) or zebrin-negative (Student’s *t*-test, *P* = 0.89; Figure 1G) cell density.

**TABLE 2:** Statistics for anterior lobules.

Age	Anterior Lobule band width						
	Zebrin-positive bands			Zebrin-negative bands			
	2_L_	1	2R	2_L_	1_L_	1_R_	2_R_
P40	*0*.*865*	*0*.*534*	*0*.*54*	*0*.*585*	0.609	*0*.*692*	*0*.*678*
P150	***0.022***	*0*.*233*	***0.0056***	0.937	*0*.*26*	0.164	*0*.*678*
P270	***0.012***	*0*.*271*	***0.002***	*0*.*269*	**0.042**	0.093	0.504
P365	***0.0009***	***0.0028***	***0.001***	0.102	**0.044**	***0.03***	0.985

*P-values are indicated for all comparisons of anterior lobule bandwidths for zebrin-positive and -negative bands at all ages studied. Mann Whitney U tests were used for italicized values, while all other comparisons were performed using Student’s t-tests. Statistically significant P-values are indicated in bold.*

**FIGURE 2:**
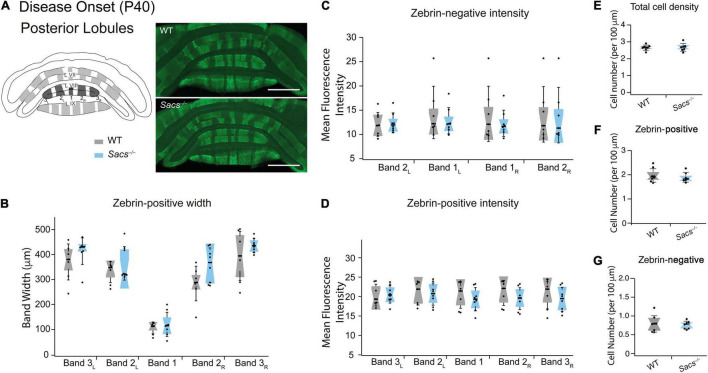
Posterior vermis zebrin expression is unaltered in disease-onset (P40) *Sacs^–/–^*mice. **(A)** Schematic (left) illustrates the coronal view of posterior lobules (L) and zebrin bands. Measurements were taken from posterior lobules VIII and IX. Sample images (right) show posterior zebrin expression in WT (top) and *Sacs^–/–^* mice (bottom). **(B)** The widths of the zebrin-positive bands in the posterior lobules were not significantly different between WT (gray) and *Sacs^–/–^* cerebellum (blue). **(C,D)** Similarly, there were no significant differences observed between the mean fluorescence intensity (arbitrary fluorescence units, a.u.) of **(C)** zebrin-positive, or **(D)** zebrin-negative bands in WT (gray) and *Sacs^–/–^* (blue) cerebellar vermis. (*N* = 6 for both WT and *Sacs^–/–^* mice. **(E–G)** There was no significant change in the total number of Purkinje cells of the posterior lobules [**(E)** the number of cells per 100 μm]. We also observed no differences in zebrin-positive **(F)** and zebrin-negative **(G)** cell densities. *N* = 4 for WT; *N* = 4 for *Sacs^–/–^*, two sections per animal; see Table 3 for *P*-values; Student’s *t*-test, *P* > 0.05 when no comparison is shown. Scale bar, 1 mm.

Zebrin-positive Purkinje cells are more prominent in posterior lobules. We predicted that zebrin expression would be unchanged in posterior *Sacs^–/–^* cerebellar vermis, since we observed no Purkinje cell firing deficits (Ady et al., 2018) and no later Purkinje cell loss (Lariviere et al., 2015, 2019) in this region. To address this, we measured zebrin expression in posterior lobules VIII and IX (Figure 2A and Supplementary Figure 3) and observed no differences in zebrin band width (Figure 2B and Table 3), or zebrin intensity in either zebrin-positive (Student’s *t*-test for all comparisons, not significantly different; zebrin-positive bands: band 3_L_: *P* = 0.878, band 2_L_: *P* = 0.89, band 1: *P* = 0.543, band 2_R_: *P* = 0.368, band 3_R_: *P* = 0.47; Figure 2C) or zebrin-negative (Student’s *t*-test for all comparisons, not significantly different; zebrin-negative bands: band 2_L_: *P* = 0.515, band 1_L_: *P* = 0.467, band 1_R_: *P* = 0.43, band 2_R_: *P* = 0.401; Figure 2D) bands from posterior lobules in WT and *Sacs^–/–^* mice. Similarly, we observed no differences in the total cell density in posterior lobules (Student’s *t*-test, *P* = 0.52; Figure 2E) or in the densities of zebrin-positive (Student’s *t*-test, *P* = 0.54; Figure 2F) or zebrin-negative (Student’s *t*-test, *P* = 0.70; Figure 2G) cells.

**TABLE 3:** Statistics for posterior lobules.

Age	Posterior Lobule band width								
	Zebrin-positive bands					Zebrin-negative bands			
	3_L_	2_L_	1	2_R_	3_R_	2_L_	1_L_	1_R_	2_R_
P40	0.076	0.10	0.483	0.063	0.524	*0.272*	0.786	*0.118*	*0.272*
0272P150	0.307	*0.12*	0.631	*0.155*	0.615	0.126	**0.035**	0.844	0.626
P270	*0.093*	***0.043***	0.434	*0.272*	*0.603*	0.138	0.303	0.723	0.974
P365	0.829	0.793	**0.018**	0.236	*0.603*	0.0514	0.50	0.375	0.437

*P-values are indicated for all comparisons of posterior lobule bandwidths for zebrin-positive and -negative bands at all ages studied. Mann Whitney U tests were used for italicized values, while all other comparisons were performed using Student’s t-tests. Statistically significant P-values are indicated in bold.*

**FIGURE 3:**
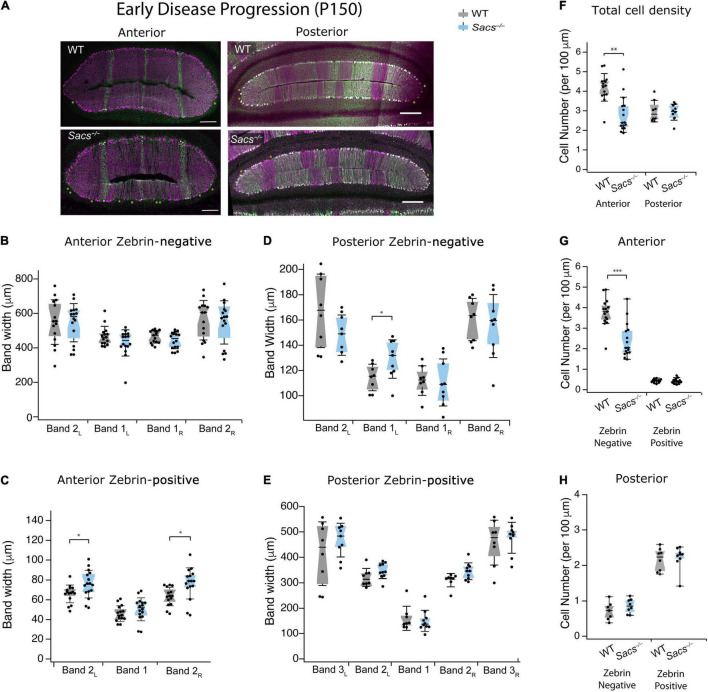
Purkinje cell death occurs primarily in zebrin-negative cells in the anterior vermis at P150. **(A)** Representative images of anterior lobules (left) and posterior lobules (right), from WT (top) and *Sacs^–/–^* (bottom) from a P150 mouse with Purkinje cells labeled with calbindin (purple) and zebrin (green). **(B)** The widths of the zebrin-negative bands in the anterior lobule were not significantly different in *Sacs^–/–^* cerebellum (blue) compared to WT (gray). **(C)** However, the widths of two of the zebrin-positive bands in the anterior lobule were significantly higher in *Sacs^–/–^* cerebellum (blue) compared to WT (gray) (*N* = 7 for WT; *N* = 8 for *Sacs^–/–^*, 2 images per animal per lobule; Mann Whitney *U* test *P < 0.05). **(D)** The width of one zebrin-negative band was elevated in *Sacs^–/–^* mice compared to WT (Band 1_L_). **(E)** The widths of zebrin-positive bands in posterior lobules were not significantly different in *Sacs^–/–^* cerebellum (blue) compared to WT (gray). **(F–H)** Total Purkinje cell density (number of cells per 100 μm) from anterior (left) and posterior (right) lobules were calculated. A significant reduction was observed in Purkinje cell density in the anterior (left), but not posterior (right) lobules at this age **(F)**, in line with previous reports (Lariviere et al., 2015). **(G)** We observed a significant reduction in the zebrin-negative (left) but not zebrin-positive (right) Purkinje cell density only anterior lobules in *Sacs^–/–^* mice compared to WT, while **(H)** neither were significantly affected in posterior lobules. (*N* = 7 for WT; *N* = 8 for *Sacs^–/–^*, 2 sections per lobule per animal; see Tables 2, 3 for *P*-values; Student’s *t*-test *P < 0.05, **P < 0.005, *P* > 0.05 when no comparison is shown, Scale bar, 200 μm.

Although Purkinje cell death is known to occur in the predominantly zebrin-negative anterior lobules of the *Sacs^–/–^* mouse cerebellum (Lariviere et al., 2015, 2019), the molecular profile of surviving neurons has not been determined in *Sacs^–/–^* mice. To ascertain this, we examined an age when Purkinje cell death is known to occur that is still relatively early during disease progression (P150; Figure 3). We used immunohistochemistry to label Purkinje cells with calbindin for total Purkinje cells, and zebrin to discern zebrin-positive (which are dual labeled) and zebrin-negative (which only express calbindin) cells. We measured the width of bands in the anterior zebrin-negative lobules and found no difference in *Sacs^–/–^* mice compared to WT (Figure 3B and Table 2). Interestingly, the width of two of the three zebrin-positive bands in the anterior lobules at P150 was slightly expanded in *Sacs^–/–^* mice compared to WT (Figure 3C and Table 2). We next measured the width of zebrin-negative (Figure 3D and Table 3) and zebrin-positive (Figure 3E and Table 3) bands in posterior lobules at P150 and found a small increase in one zebrin-negative band (band 1_L_; Figure 3D and Table 3), with no other statistically significant differences in *Sacs^–/–^* mice posterior bands.

To determine if changes in band width reflect changes in Purkinje cell density at P150, we next measured this in WT and *Sacs^–/–^* mice (Figure 3F). We found that the total density of calbindin-labeled Purkinje cell somata was reduced in anterior lobules in P150 *Sacs^–/–^* mice, consistent with previous reports (Student’s *t*-test, *P* < 0.001; Figure 3F) (Lariviere et al., 2015). To determine whether this reduction was observed equally in zebrin-positive and -negative Purkinje cells, we then examined cells of each molecular profile separately. We observed a significant reduction in anterior lobule zebrin-negative Purkinje cells (Student’s *t*-test, *P* < 0.0001; Figure 3G, left). Although the number of zebrin-positive cells in anterior lobules is low (Figure 3A, left), we did not observe any significant differences in their density in *Sacs^–/–^* mice (Student’s *t*-test, *P* = 0.68; Figure 3G). Since cell death has not been reported in posterior lobules in *Sacs^–/–^* mice (Lariviere et al., 2015, 2019), we expected no differences in the proportion of zebrin-positive and -negative Purkinje cells there. Consistent with this, we observed that the densities of both zebrin-positive and -negative cells in posterior lobules were not significantly different in P150 *Sacs^–/–^* mice (Student’s *t*-test for all measurements: *P* = 0.82 for total cell density; *P* = 0.44 for zebrin-negative cell density; and *P* = 0.70 for zebrin-positive cell density; Figure 3H). These results suggest that Purkinje cell death occurs primarily in zebrin-negative neurons in anterior lobules at an early stage of disease progression (P150) and that although zebrin-positive bands expand, they appear to do so to occupy the space left by the zebrin-negative cell death.

**FIGURE 4:**
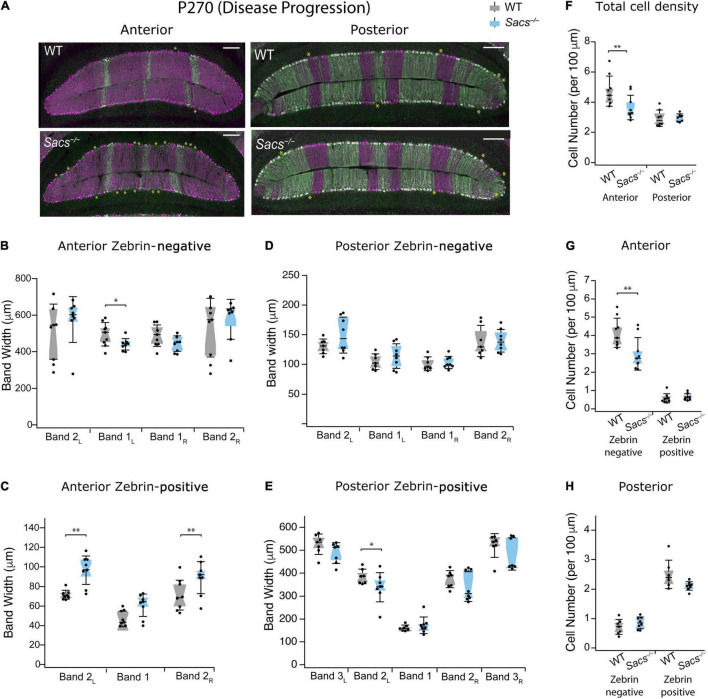
Patterned cell death persists as the disease progresses in zebrin-negative Purkinje cells in anterior vermis in P270 mice. **(A)** Representative images of anterior lobules (left) and posterior lobules (right), from WT (top) and *Sacs^–/–^* (bottom) from a P270 mouse with Purkinje cells labeled with calbindin (purple) and zebrin (green). Asterisks indicate gaps in Purkinje cells. **(B)** The widths of three of the four zebrin-negative bands in the anterior lobule were not significantly different in *Sacs^–/–^* cerebellum (blue) compared to WT (gray) although band 1_L_ was moderately reduced. **(C)** The widths of two zebrin-positive bands (2_L_ and 2_R_) in the anterior lobule were significantly increased in *Sacs^–/–^* cerebellum (blue) compared to WT (gray). **(D,E)** In posterior lobules at P365, neither **(D)** zebrin-negative nor **(E)** most of the zebrin-positive band width was affected in *Sacs^–/–^* cerebellum (blue) compared to WT (gray). **(F)** Total Purkinje cell density (cells per 100 μm) showed a significant reduction in anterior (left) but not posterior lobules (right). **(G)** In anterior lobules, this reduction was only observed in zebrin-negative (left) but not zebrin-positive (right) Purkinje cells **(H)**. However, in posterior lobules, neither zebrin-negative (left) nor zebrin-positive (right) cells showed significant differences. *N* = 4 for WT; *N* = 4 for *Sacs^–/–^*, 2 sections per lobule per animal; see Tables 2, 3 for *P*-values; Student’s *t*-test used for normally distributed and Mann Whitney *U* test used for non-normally distributed data; *P < 0.05, **P < 0.005, *P* > 0.05 if no comparison is shown. Scale bar, 200 μm.

ARSACS is a progressive neurodegenerative disease, and in mouse models, increased levels of Purkinje cell death have been observed with aging (Lariviere et al., 2015, 2019). Therefore, we wondered whether advanced stages of disease progression extend cell death to include previously resilient zebrin-positive cells. To determine this, we measured zebrin-positive and -negative Purkinje cells in anterior and posterior lobules at advancing disease stages. First, we examined P270 (9 months; Figure 4A). We found that in anterior lobules, one zebrin-negative band was reduced, while two were unchanged (Figure 4B and Table 2) and two of the three zebrin-positive bands were wider in *Sacs^–/–^* mice (Figure 4C and Table 2), just as we observed at P150. In posterior lobules, however, there was only a small reduction observed in one zebrin-positive band (band 2_L_; Figure 4E and Table 3), with no other differences were observed in the remaining zebrin-positive and zebrin-negative bands (Figures 4D,E and Table 3). Similar to our findings at P150, the total cell density was reduced in anterior but not posterior lobules in *Sacs^–/–^* mice (Student’s *t*-test, *P* = 0.02; Figure 4F), which was reflected in a reduction in zebrin-negative, but not zebrin-positive cell density in anterior lobules (Student’s *t*-test for both measurements; zebrin-negative cell density: *P* = 0.01; zebrin-positive density: *P* = 0.52; Figure 4G). However, no differences in total zebrin-positive or -negative cells were observed in posterior lobules (Student’s *t*-test for all comparisons; total: *P* = 0.13; zebrin-negative: *P* = 0.26; zebrin-positive: *P* = 0.08; Figure 4H).

**FIGURE 5:**
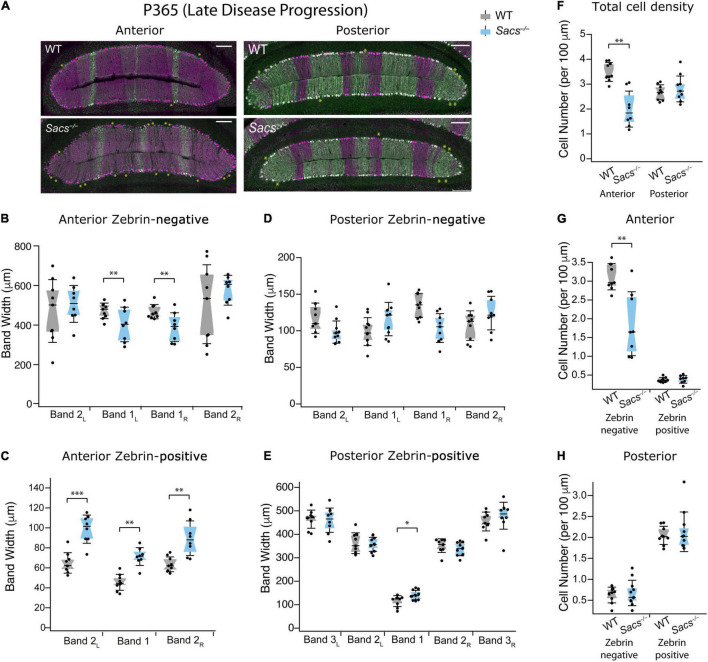
Patterned cell death at late disease progression (P365) is restricted to zebrin-negative Purkinje cells in the anterior vermis. **(A)** Representative images of anterior lobules (left) and posterior lobules (right), from WT (top) and *Sacs^–/–^* (bottom) from a P365 mouse with Purkinje cells labeled with calbindin (purple) and zebrin (green). Asterisks indicate gaps in Purkinje cells. **(B)** The widths of zebrin-negative bands 1_L_ and 1_R_ were significantly reduced in *Sacs^–/–^* anterior cerebellum (blue) compared to WT (gray). **(C)** The widths of all 3 zebrin-positive bands in the anterior cerebellum were significantly increased in *Sacs^–/–^* (blue) compared to WT (gray). **(D,E)** However, in posterior lobules at P365, neither **(D)** zebrin-negative nor **(E)** most of zebrin-positive band width was affected in *Sacs^–/–^* cerebellum (blue) compared to WT (gray). **(F)** Total Purkinje cell density (number of cells per 100 μm) showed a significant reduction anterior (left) but not posterior lobules (right). **(G)** In anterior lobules, this reduction was only observed in zebrin-negative (left) but not zebrin-positive (right) Purkinje cells **(H)**. However, in posterior lobules, neither zebrin-negative (left) nor zebrin-positive (right) cells showed significant differences. *N* = 4 for WT; *N* = 4 for *Sacs^–/–^*, 2 sections per lobule per animal; see Tables 2, 3 for *P*-values; Student’s *t*-test used for normally distributed and Mann Whitney *U* test used for non-normally distributed data; *P < 0.05, ^**^P < 0.005, ^***^P < 0.001, *P* > 0.05 if no comparison is shown. Scale bar, 200 μm.

Finally, we examined an even more advanced stage of disease progression at 1 year (P365; Figure 5A) and found results similar to those at P150 and P270, but more severe. We now observed that two out of four anterior zebrin-negative bands were reduced in *Sacs^–/–^* mice compared to WT (Figure 5B and Table 2), and this was accompanied by significant increases in the band width of all three anterior zebrin-positive bands in *Sacs^–/–^* mice at P365 (Figure 5C and Table 2), suggesting that Purkinje cells become more disorganized in anterior lobules as the disease progresses. However, restructuring was predominantly observed in anterior lobules, as even at this later disease stage, posterior bandwidths appeared largely normal in *Sacs^–/–^* mice for zebrin-negative (Figure 5D and Table 3) and most zebrin-positive (Figure 5E and Table 3) bands, other than a small increase in zebrin-positive band 1 (Figure 5E and Table 3). The density of Purkinje cells in *Sacs^–/–^* mice at P365 was again only affected in anterior, but not posterior lobules (Student’s *t*-test for both comparisons; anterior: *P* = 0.003; posterior: *P* = 0.48; Figure 5F). Similar to what we observed at earlier ages, this change appeared to arise predominantly from changes in anterior zebrin-negative, but not zebrin-positive cell density (Student’s *t*-test for both comparisons; zebrin-negative cell density: *P* = 0.004; zebrin-positive cell density: *P* = 0.75; Figure 5G). In agreement with results from overall posterior cell density at P365 (Figure 5F), no change in posterior zebrin-negative and -positive cell densities was observed in *Sacs^–/–^* mice at P365 (Student’s *t*-test for both comparisons, zebrin-negative cell density: *P* = 0.67; zebrin-positive cell density: *P* = 0.60; Figure 5H). These results agree with our hypothesis that cell death is largely limited to anterior lobules even at relatively advanced disease stages and that this is manifested by changes in cell density of zebrin-negative cells. However, although no changes in zebrin-positive numbers are observed, anterior lobules become more disorganized at P365 in *Sacs^–/–^* mice, likely because zebrin-positive bands expand into space where zebrin-negative cells have died.

**FIGURE 6:**
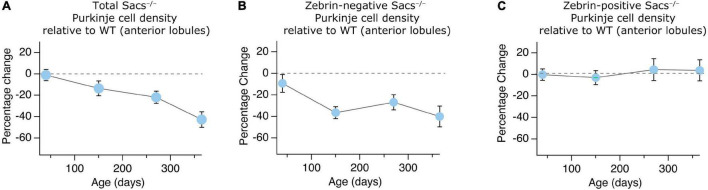
Gradual anterior Purkinje cell loss across ages in *Sacs^–/–^* mice. **(A–C)** The proportion of Purkinje cells in anterior lobules (% change *Sacs^–/–^* relative to WT) at each age is plotted. We observed **(A)** a gradual decrease in total Purkinje cell number in anterior lobules that is similar to **(B)** the time course of loss observed in zebrin-negative cells in anterior lobules. **(C)** However, there are no proportional changes in zebrin-positive cell number across ages in *Sacs^–/–^* mice. Data are represented as averages ± SEM.

Given that we have examined the progression of Purkinje cell loss in a mouse model of ARSACS over time, we wondered whether this represented change arising from aging or from disease progression. We compared the change in anterior lobule Purkinje cells across time, from P40 to P365, which allows us to normalize to WT cells, and thus graphically represent the contribution arising exclusively from disease progression. We observed a gradual decrease of Purkinje cell numbers in anterior lobules (Figure 6A), which was reflected in the proportion of zebrin-negative cells over time (Figure 6B), while the proportion of zebrin-positive cells remained remarkably similar at all ages (Figure 6C).

### Patterned Loss of Purkinje Cell Puncta Made Onto Large Cells in the Cerebellar Nuclei

Purkinje cell death is one of the most prominent pathophysiological changes in both ARSACS brains and animal models of ARSACS, but other changes have also been observed. For instance, although the numbers of cells in the CN of the cerebellum are unaltered in *Sacs^–/–^* cerebellum, the number of puncta, likely reflecting the number of synaptic inputs onto these cells, is reduced at the onset of disease in *Sacs^–/–^* mice (Ady et al., 2018). Changes in the innervation of target neurons in the CN will likely have a profound impact on cerebellar function. This raises the question of whether this reduction arises from changes in numbers of inputs from all Purkinje cells or whether specific subsets of Purkinje cells also show preferential changes in their innervation of the CN?

**FIGURE 7:**
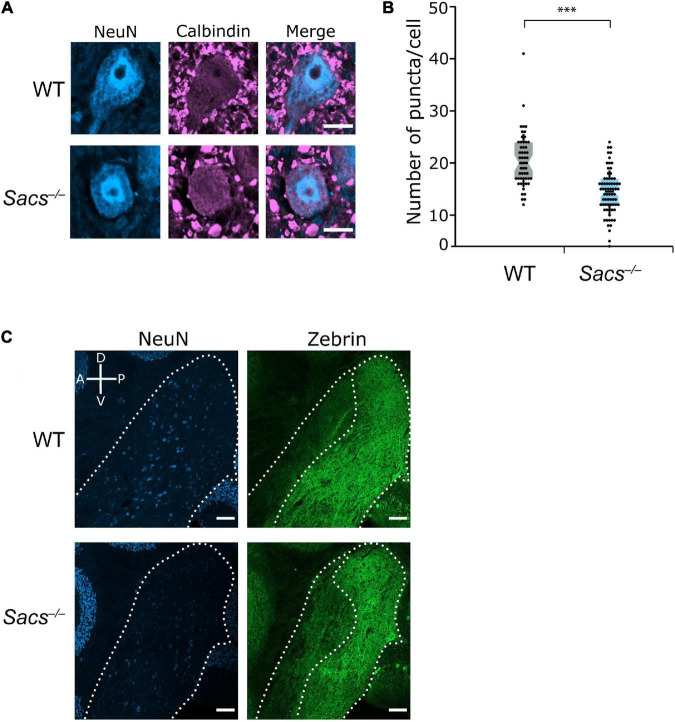
Reduction in Purkinje cell puncta in cerebellar nuclei (CN) in *Sacs^–/–^* mice. **(A)** Representative images of large neurons (>15 μm diameter) in the CN with calbindin-positive puncta as a measure of Purkinje cell inputs. Scale bar, 10 μm. **(B)** The number of calbindin-positive puncta onto large cells in the CN is significantly lower in *Sacs^–/–^* mice, data shown per cell (Student’s *t*-test, *P* < 0.000001). **(C)** Representative images show that zebrin-positive Purkinje cell innervation is predominantly restricted to the posterior half of the CN in both WT and *Sacs^–/–^* mice. The compass shows dorsal-ventral and anterior-posterior axes. Scale bar, 100 μm. ^***^P < 0.0001.

We first determined whether the decrease in the number of puncta, which we used as a proxy for the number of potential Purkinje cell terminals innervating the CN neurons, that we previously observed at disease onset (Ady et al., 2018), was observed as the disease progressed (at P90). We measured the number of calbindin-positive Purkinje cell puncta made onto large projection neurons in the fastigial and interposed nuclei (Figure 7A) since these receive innervation from the vermis (Voogd and Ruigrok, 2004). We found that the number of puncta was reduced in *Sacs^–/–^* mice (Student’s *t-*test; *P* < 0.0001; Figures 7A,B), similar to what is observed at earlier ages (Ady et al., 2018). We have previously demonstrated that in the CN of both WT and *Sacs^–/–^* mice, there is a high degree of colocalization between calbindin-positive puncta and puncta that are stained with VGAT at P40 (Ady et al., 2018). This indicates that these previously identified calbindin-positive puncta are likely functional GABAergic Purkinje cell terminals. We found a high degree of co-labeling in WT and *Sacs^–/–^* mice at P90 (Supplementary Figure 2). Thus, the reduction in the number of calbindin-positive puncta in the CN of ARSACS mice at P90 appears to reflect a loss of functional Purkinje cell presynaptic terminals rather than a shift in the proportion of inputs arising from Purkinje cell terminals compared to other inputs.

It has previously been described that the pattern of zebrin projections respects the anterior – posterior (or rostral – caudal) division of the CN (Hawkes and Leclerc, 1986; Sugihara, 2011), with the anterior regions of the nuclei receiving predominantly zebrin-negative input and posterior nuclei receiving predominantly zebrin-positive input (Sugihara, 2011). We observed this division in both WT and *Sacs^–/–^* mice (Figure 7C). Thus, to determine whether changes were made in the number of zebrin-positive or -negative puncta in the CN, we analyzed neurons with respect to their anterior – posterior location within both CN nuclei.

**FIGURE 8:**
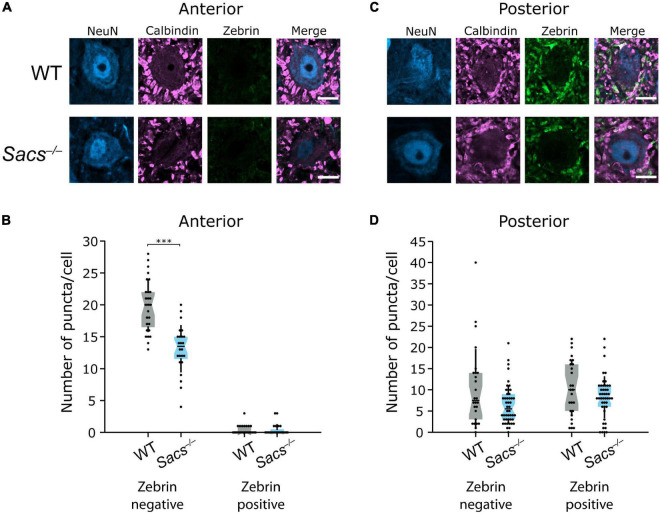
Reduced innervation of CN neurons is restricted to anterior zebrin-negative puncta in *Sacs^–/–^* mice. **(A)** Representative images of large (>15 μm) CN neurons from the anterior interposed nucleus, with calbindin-positive puncta from Purkinje cells but minimal zebrin-positive puncta. **(B)** Anterior cells in the CN received significantly fewer zebrin-negative Purkinje cell (calbindin only) puncta in *Sacs^–/–^* mice compared to WT (left; Student’s *t*-test, *P* < 0.000001). However, neither WT nor *Sacs^–/–^* anterior CN neurons received significant input from zebrin-positive Purkinje cells and there were no significant differences between them (Mann Whitney *U* test, *P* = 0.81). **(C)** Representative images of CN neurons from the posterior interposed nucleus. **(D)** Both WT and *Sacs^–/–^* CN neurons received input from both zebrin-positive and -negative puncta and there was no difference in either the numbers of zebrin-negative (left) or -positive (right) puncta between groups (zebrin-negative: Mann Whitney *U* test, *P* = 0.146, zebrin-positive: Student’s *t*-test, *P* = 0.163). ^***^P < 0.0001; *P* > 0.05 when no comparison is shown. Scale bar = 10 μm.

In anterior CN, which receives largely zebrin-negative Purkinje cell innervation, we observed a reduction in the number of zebrin-negative puncta onto large CN neurons compared to WT (WT: *N* = 4; *Sacs^–/–^ N* = 3; Student’s *t*-test, *P* < 0.0001; Figures 8A,B). The number of zebrin-positive Purkinje cell puncta in anterior CN was low in both WT and *Sacs^–/–^* mice and did not significantly differ between genotype (Mann Whitney *U* test, *P* = 0.81; Figures 8A,B), which suggests that no major rewiring of puncta occurs from zebrin-positive cells. Interestingly, although the total number of zebrin-negative puncta made onto neurons was lower in posterior CN compared to anterior CN, there was no significant reduction in the number of zebrin-negative puncta in *Sacs^–/–^* cerebellum compared to WT (Mann Whitney *U* test, *P* = 0.15; Figures 8C,D). We also observed unchanged numbers of zebrin-positive Purkinje cell puncta in posterior CN in *Sacs^–/–^* mice (Student’s *t*-test, *P* = 0.16; Figures 8C,D). These data suggest that reduction in the innervation of targets in the CN by Purkinje cell axons is predominantly arising from alterations in zebrin-negative Purkinje cells in the anterior vermis. Taken together, our results are consistent with a specific population of Purkinje cells, which do not express zebrin and reside in anterior vermis, that are uniquely vulnerable to loss of sacsin. Vulnerable cells display firing deficits (Ady et al., 2018), show reduced innervation of downstream targets (although Purkinje cells in other projection regions may also be affected in this altered innervation pattern, Figures 8A,B) and also are vulnerable to Purkinje cell death as the disease progresses (Figures 3–6).

## Discussion

In this study, we show that the patterned Purkinje cell death that has been previously described in *Sacs^–/–^* mice (Lariviere et al., 2015, 2019) occurs predominantly in cells in the anterior vermis that do not express the molecule zebrin, even at late disease stages. Although these cells eventually die, their development appears normal, as the zebrin patterning that arises during development appears largely unaffected in young adult mice prior to Purkinje cell death. Projections from Purkinje cells to target large neurons in the CN also show a pattern of degeneration: reduced synaptic innervation is observed only among zebrin-negative calbindin-positive puncta in anterior CN, but neither zebrin-negative nor zebrin-positive puncta appear affected in posterior CN. These findings support the hypothesis that zebrin-negative Purkinje cells residing in the anterior vermis are uniquely susceptible to cell death in ARSACS.

Patterned Purkinje cell death has been observed in several different diseases and conditions. Sarna and Hawkes suggested that four distinct patterned cell death motifs existed (Sarna and Hawkes, 2003): (1) zebrin-negative cells were uniquely susceptible to cell death, (2) zebrin-positive cells were uniquely susceptible to cell death, (3) cell death is not random, but does not respect a particular molecular profile, or (4) Purkinje cells die randomly. While zebrin-negative cells appear to be uniquely affected in *Sacs^–/–^* mice, it appears that not all zebrin-negative cells are susceptible, only those residing in anterior lobules. It is possible that in addition to the lack of zebrin expression, there are additional molecules that can be used to characterize these cells. Indeed, there exist molecules that are expressed in subsets of Purkinje cells in patterns that are more reminiscent of the patterning of degeneration we observe, such as PLCβ4 (Sarna et al., 2006), and it is likely that heterogeneity exists even within the zebrin bands that we describe. The predominantly zebrin-negative anterior-vermis degeneration observed here is reminiscent of several other forms of ataxia that show similar patterns of degeneration (Sarna and Hawkes, 2003), including SCA1 (White et al., 2021). It is possible that diseases that share similar patterns of cell degeneration may share common pathophysiological pathways (Niewiadomska-Cimicka et al., 2021), which suggests that common treatment strategies may be pursued for these disparate diseases.

Although we observe no significant differences in zebrin-positive Purkinje cells in anterior lobules since their density is low, it is reasonable to question whether anterior zebrin-positive cells do also degenerate, but their proportion is simply too small to detect. Given that we have queried zebrin-positive cell density at four different ages, and observed virtually identical results across these ages (Figure 6C), even while cell death increases in *Sacs^–/–^* mice overall to ∼40% of WT levels (Figure 6A), we feel that this is unlikely, although still possible. We do see systematic increases in the width of zebrin-positive bands as zebrin-negative cells die, suggesting that some rearrangement does occur in the Purkinje cell layer. However, the most parsimonious explanation for our data is that zebrin-positive cells are resilient to cell death in anterior lobules.

We have previously observed that CN neurons showed reduced innervation by Purkinje cell puncta in P40 *Sacs^–/–^* mice, before cell death in Purkinje cells is observed (Figures 1, 2; Ady et al., 2018). Purkinje cell projections to the CN are not randomly organized, but cluster into different zones with different molecular profiles (Chung et al., 2009), and CN neurons receiving input from predominantly zebrin-negative Purkinje cells fire at higher frequencies *in vivo* than those that receive input predominantly from zebrin-positive Purkinje cells. Therefore, the preferential loss of either zebrin-negative or -positive innervation of the CN likely leads to a drastic alteration in the output of the cerebellar circuit (Beekhof et al., 2021). Notably, there is very little zebrin-positive innervation in the anterior portions of the nuclei (Hawkes and Leclerc, 1986; Sugihara, 2011). Given that firing deficits (Ady et al., 2018) and cell death are predominantly in anterior lobules (Figures 3–5; Lariviere et al., 2015), we wondered whether there were changes in the innervation of the CN that we had hitherto missed in *Sacs^–/–^* mice, since we had not explored the molecular profile of inputs, nor where the neurons we examined were located within the CN. We found that the innervation deficits that we had previously reported (Ady et al., 2018) arose exclusively due to a reduction in zebrin-negative innervation of anterior CN neurons, with no differences observed for neurons in posterior CN in either zebrin-negative or -positive inputs. This suggests that both zebrin identity and positional information help determine the deficits observed in *Sacs^–/–^* mice. Further molecular subdivision of zebrin-negative neurons may exist, which might shed light on pathophysiological pathways in the Purkinje cells affected in ARSACS.

We have previously reported that sacsin is expressed in cerebellar Purkinje cells (Lariviere et al., 2019), where we did not observe any visible patterning in its distribution. Our results suggest that it is a particular subset of Purkinje cells that are vulnerable to loss of sacsin in ARSACS and that these vulnerable cells, which do not express zebrin and are located in anterior lobules in the cerebellar vermis, also are likely those that display firing deficits (Ady et al., 2018). However, it is possible that the reduced innervation from zebrin-negative puncta in anterior CN (Figure 6) arises from these anterior zebrin-negative Purkinje cells, although zebrin-negative Purkinje cells in other regions may contribute as well. Our data caution that it is essential to consider both the location and the molecular identity of Purkinje cells when studying the disease-causing changes in ARSACS, and likely in other cerebellar disorders since zebrin-negative cells in posterior lobules appear to be resilient. Perhaps by examining the expression pattern of several molecular markers that display unique patterned expression, we will identify a molecular characterization that uniquely identifies the susceptible population of Purkinje cells in ARSACS. Understanding the molecular characteristics that give rise to this vulnerability may contribute to understanding the pathophysiology of ARSACS.

## Data Availability Statement

The raw data supporting the conclusions of this article will be made available by the authors, without undue reservation.

## Ethics Statement

The animal study was reviewed and approved by McGill University Animal Care Committee.

## Author Contributions

RM and AW conceived and designed the manuscript, supervised the study. BT, AC, AS, MR, KV-L, and FC performed the experiments and analyzed the data. BT, AC, RM, and AW wrote the manuscript. All the authors agreed to be accountable for the content of the study.

## Conflict of Interest

The authors declare that the research was conducted in the absence of any commercial or financial relationships that could be construed as a potential conflict of interest.

## Publisher’s Note

All claims expressed in this article are solely those of the authors and do not necessarily represent those of their affiliated organizations, or those of the publisher, the editors and the reviewers. Any product that may be evaluated in this article, or claim that may be made by its manufacturer, is not guaranteed or endorsed by the publisher.

## Footnotes

1
https://mouse.brain-map.org/static/atlas

## References

[B1] Ady V. Marquez B. T Nath M. Chang P. K. Hui J. Cook A. C. (2018). Altered Synaptic and Intrinsic properties of cerebellar Purkinje cells in a mouse model of ARSACS. *J. Physiol.* 596 4253–4267. 29928778 10.1113/JP275902 PMC6117548

[B2] Apps R. Hawkes R. (2009). Cerebellar cortical organization: a one-map hypothesis. *Nat. Rev. Neurosci.* 10 670–681. 19693030 10.1038/nrn2698

[B3] Bailey K. Rahimi Balaei M. Mannan A. Del Bigio M. R. Marzban H. (2014). Purkinje cell compartmentation in the cerebellum of the lysosomal Acid phosphatase 2 mutant mouse (nax - naked-ataxia mutant mouse). *PLoS One* 9:e94327. 24722417 10.1371/journal.pone.0094327 PMC3983142

[B4] Beekhof G. C. Gornati S. V. Canto C. B. Libster A. M. Schonewille M. De Zeeuw C. I. (2021). Activity of Cerebellar Nuclei Neurons Correlates with ZebrinII Identity of Their Purkinje Cell Afferents. *Cells* 10:2686. 34685666 10.3390/cells10102686 PMC8534335

[B5] Bouchard J. P. Barbeau A. Bouchard R. Bouchard R. W. (1978). Autosomal recessive spastic ataxia of Charlevoix-Saguenay. *Can J. Neurol. Sci.* 5 61–69. 647499

[B6] Bouchard J. P. Richter A. Mathieu J. Brunet D. Hudson T. J. Morgan K. (1998). Autosomal recessive spastic ataxia of Charlevoix-Saguenay. *Neuromuscul. Disord.* 8 474–479. 9829277 10.1016/s0960-8966(98)00055-8

[B7] Brochu G. Maler L. Hawkes R. (1990). Zebrin II: a polypeptide antigen expressed selectively by Purkinje cells reveals compartments in rat and fish cerebellum. *J. Compar. Neurol.* 291 538–552. 10.1002/cne.902910405 2329190

[B8] Chung S. H. Marzban H. Hawkes R. (2009). Compartmentation of the cerebellar nuclei of the mouse. *Neuroscience* 161 123–138. 19306913 10.1016/j.neuroscience.2009.03.037

[B9] Duncan E. J. Lariviere R. Bradshaw T. Y. Longo F. Sgarioto N. Hayes M. J. (2017). Altered organization of the intermediate filament cytoskeleton and relocalization of proteostasis modulators in cells lacking the ataxia protein sacsin. *Hum. Mol. Genet.* 26 3130–3143. 28535259 10.1093/hmg/ddx197 PMC5886247

[B10] Engert J. C. Berube P. Mercier J. Dore C. Lepage P. Ge B. (2000). ARSACS a spastic ataxia common in northeastern Quebec, is caused by mutations in a new gene encoding an 11.5-kb ORF. *Nat. Genet.* 24 120–125. 10655055 10.1038/72769

[B11] Gentil B. J. Lai G. T. Menade M. Lariviere R. Minotti S. Gehring K. (2019). Sacsin, mutated in the ataxia ARSACS, regulates intermediate filament assembly and dynamics. *FASEB J.* 33 2982–2994. 30332300 10.1096/fj.201801556R

[B12] Girard M. Lariviere R. Parfitt D. A. Deane E. C. Gaudet R. Nossova N. (2012). Mitochondrial dysfunction and Purkinje cell loss in autosomal recessive spastic ataxia of Charlevoix-Saguenay (ARSACS). *Proc. Natl. Acad. Sci. U S A* 109 1661–1666. 22307627 10.1073/pnas.1113166109 PMC3277168

[B13] Hawkes R. (2014). Purkinje cell stripes and long-term depression at the parallel fiber-Purkinje cell synapse. *Front. Syst. Neurosci.* 8:41. 24734006 10.3389/fnsys.2014.00041 PMC3975104

[B14] Hawkes R. Leclerc N. (1986). Immunocytochemical demonstration of topographic ordering of Purkinje cell axon terminals in the fastigial nuclei of the rat. *J. Comparat. Neurol.* 244 481–491. 10.1002/cne.902440406 3514691

[B15] Lariviere R. Gaudet R. Gentil B. J. Girard M. Conte T. C. Minotti S. (2015). Sacs knockout mice present pathophysiological defects underlying autosomal recessive spastic ataxia of Charlevoix-Saguenay. *Hum. Mol. Genet.* 24 727–739. 25260547 10.1093/hmg/ddu491 PMC4291249

[B16] Lariviere R. Sgarioto N. Marquez B. T. Gaudet R. Choquet K. McKinney R. A. (2019). Sacs R272C missense homozygous mice develop an ataxia phenotype. *Mol. Brain* 12:19. 30866998 10.1186/s13041-019-0438-3 PMC6416858

[B17] Larouche M. Hawkes R. (2006). From clusters to stripes: the developmental origins of adult cerebellar compartmentation. *Cerebellum* 5 77–88. 16818382 10.1080/14734220600804668

[B18] Marzban H. Hawkes R. (2011). On the architecture of the posterior zone of the cerebellum. *Cerebellum* 10 422–434. 20838950 10.1007/s12311-010-0208-3

[B19] Miyazaki T. Yamasaki M. Hashimoto K. Yamazaki M. Abe M. Usui H. (2012). Cav2.1 in cerebellar Purkinje cells regulates competitive excitatory synaptic wiring, cell survival, and cerebellar biochemical compartmentalization. *J. Neurosci.* 32 1311–1328. 22279216 10.1523/JNEUROSCI.2755-11.2012 PMC6796260

[B20] Niewiadomska-Cimicka A. Doussau F. Perot J.-B. Roux M. J. Keime C. Hache A. (2021). SCA7 mouse cerebellar pathology reveals preferential downregulation of key Purkinje cell-identity genes and shared disease signature with SCA1 and SCA2. *J. Neurosci.* 2021 1882–1820. 10.1523/JNEUROSCI.1882-20.2021 PMC8260160 33888607

[B21] Parfitt D. A. Michael G. J. Vermeulen E. G. Prodromou N. V. Webb T. R. Gallo J. M. (2009). The ataxia protein sacsin is a functional co-chaperone that protects against polyglutamine-expanded ataxin-1. *Hum. Mol. Genet.* 18 1556–1565. 19208651 10.1093/hmg/ddp067 PMC2667285

[B22] Perkins E. M. Clarkson Y. L. Suminaite D. Lyndon A. R. Tanaka K. Rothstein J. D. (2018). Loss of cerebellar glutamate transporters EAAT4 and GLAST differentially affects the spontaneous firing pattern and survival of Purkinje cells. *Hum. Mol. Genet.* 27 2614–2627. 29741614 10.1093/hmg/ddy169 PMC6049029

[B23] Sarna J. R. Hawkes R. (2003). Patterned Purkinje cell death in the cerebellum. *Prog. Neurobiol.* 70 473–507. 14568361 10.1016/s0301-0082(03)00114-x

[B24] Sarna J. R. Hawkes R. (2011). Patterned Purkinje cell loss in the ataxic sticky mouse. *Eur. J. Neurosci.* 34 79–86. 21645134 10.1111/j.1460-9568.2011.07725.x

[B25] Sarna J. R. Marzban H. Watanabe M. Hawkes R. (2006). Complementary stripes of phospholipase Cbeta3 and Cbeta4 expression by Purkinje cell subsets in the mouse cerebellum. *J. Comparat. Neurol.* 496 303–313. 10.1002/cne.20912 16566000

[B26] Sawada K. Kalam Azad A. Sakata-Haga H. Lee N. S. Jeong Y. G. Fukui Y. (2009). Striking pattern of Purkinje cell loss in cerebellum of an ataxic mutant mouse, tottering. *Acta Neurobiol. Exp.* 69 138–145. 10.55782/ane-2009-1736 19325647

[B27] Sugihara I. (2011). Compartmentalization of the deep cerebellar nuclei based on afferent projections and aldolase C expression. *Cerebellum* 10 449–463. 20981512 10.1007/s12311-010-0226-1

[B28] Voogd J. Ruigrok T. J. (2004). The organization of the corticonuclear and olivocerebellar climbing fiber projections to the rat cerebellar vermis: the congruence of projection zones and the zebrin pattern. *J. Neurocytol.* 33 5–21. 15173629 10.1023/B:NEUR.0000029645.72074.2b

[B29] Wadiche J. I. Jahr C. E. (2005). Patterned expression of Purkinje cell glutamate transporters controls synaptic plasticity. *Nat. Neurosci.* 8 1329–1334. 16136036 10.1038/nn1539

[B30] White J. J. Arancillo M. Stay T. L. George-Jones N. A. Levy S. L. Heck D. H. (2014). Cerebellar zonal patterning relies on Purkinje cell neurotransmission. *J. Neurosci.* 34 8231–8245. 24920627 10.1523/JNEUROSCI.0122-14.2014 PMC4051975

[B31] White J. J. Bosman L. W. J. Blot F. G. C. Osorio C. Kuppens B. W. Krijnen W. (2021). Region-specific preservation of Purkinje cell morphology and motor behavior in the ATXN1[82Q] mouse model of spinocerebellar ataxia 1. *Brain Pathol.* 2021:e12946. 10.1111/bpa.12946 PMC8412070 33724582

[B32] Xiao J. Cerminara N. L. Kotsurovskyy Y. Aoki H. Burroughs A. Wise A. K. (2014). Systematic regional variations in Purkinje cell spiking patterns. *PLoS* O*ne* 9:e105633. 10.1371/journal.pone.0105633 PMC4140808 25144311

[B33] Zhou H. Lin Z. Voges K. Ju C. Gao Z. Bosman L. W. (2014). Cerebellar modules operate at different frequencies. *Elife* 3:e02536. 24843004 10.7554/eLife.02536 PMC4049173

