# Supplementary material for: Molecular Identity and Location Influence Purkinje Cell Vulnerability in Autosomal-Recessive Spastic Ataxia of Charlevoix-Saguenay Mice
Source: Front Cell Neurosci. 2021 Dec 14;15:707857. doi: 10.3389/fncel.2021.707857 (PMC8712330; doi:10.3389/fncel.2021.707857)
Supplement: Supplementary file 1 [file Data_Sheet_1.pdf]

Molecular identity and location dictate Purkinje cell susceptibility in ARSACS mice

Supplementary Information

Brenda Toscano Márquez[1], Anna A. Cook[1], Max Rice[1], Alexia Smileski[1], Kristen Vieira-Lomasney[1], François Charron[2], R. Anne McKinney[2]* and Alanna J. Watt[1]*

[1]Department of Biology, McGill University, Montreal, QC Canada
[2]Department of Pharmacology and Therapeutics, McGill University, Montreal, QC Canada

Includes: 3 Supplementary Figures

Supplementary Figures and Figure Legends

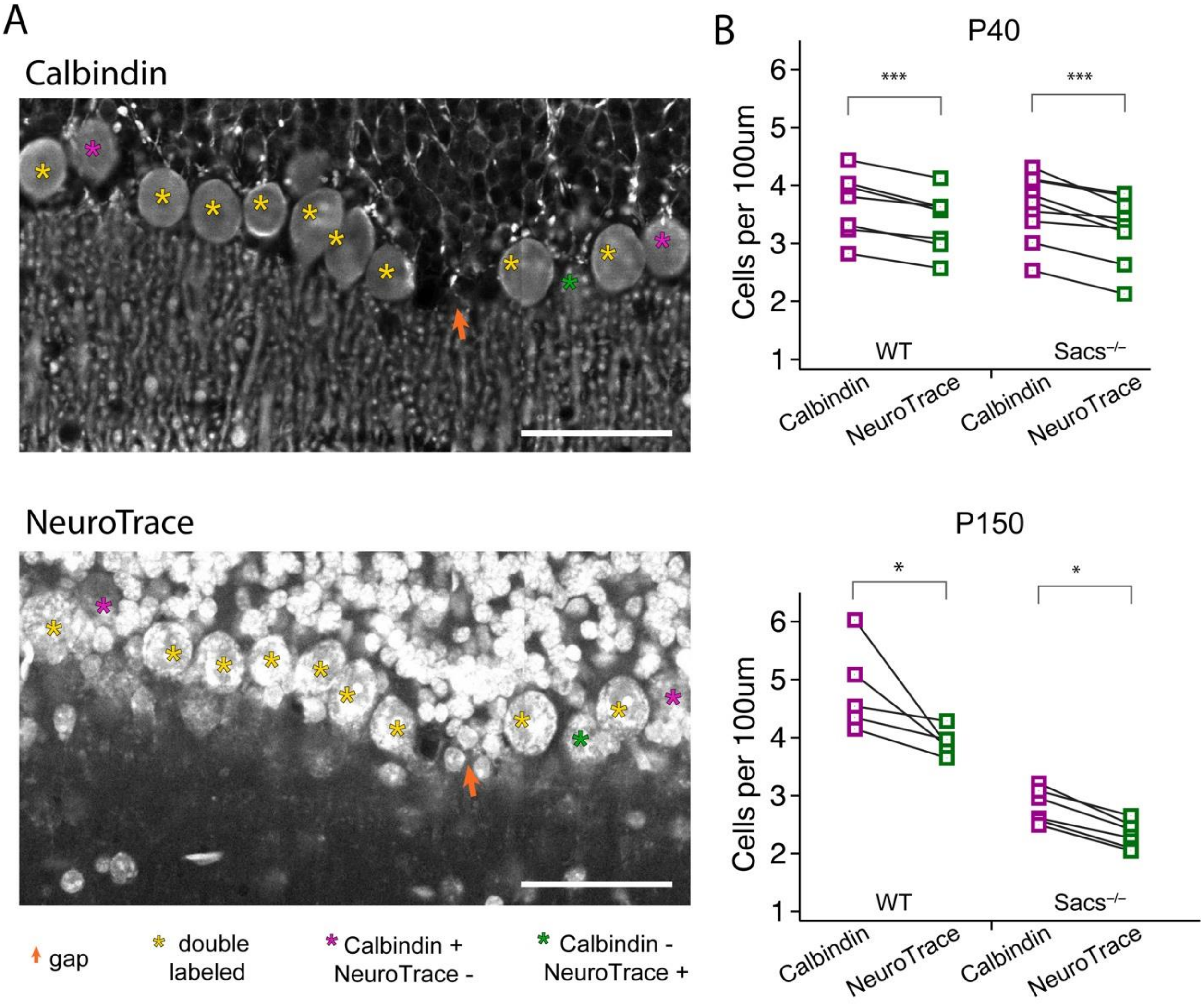

Supplemental Figure 1. Validation of Calbindin as a marker for Purkinje cells.
(A) Representative image of immunohistochemistry for Calbindin (top) and NeuroTrace, a fluorescent Nissl stain (bottom) in a cerebellar section of an anterior lobule. Cells were counted in each channel separately then compared. Most of the Purkinje cells show positive labeling for both, Calbindin and NeuroTrace (yellow asterisk). However, a few cells were Calbindin positive but were not detected with NeuroTrace (magenta asterisk). NeuroTrace labels many cell types, including granular cells and interneurons, which could occasionally block detection of a Purkinje cell. The gaps occasionally observed in the Purkinje cell layer with Calbindin, were also evident with NeuroTrace staining (orange arrow, although small, non-Purkinje cells are present). (**B)** Quantification of the density of Calbindin and NeuroTrace cells in sections of the anterior lobule of WT and *Sacs*$^{-/-}$ animals at P40 (before cell loss) and at P150 (where Purkinje cell loss is observed). More cells were consistently detected with Calbindin than Neurotrace in all conditions, arguing that there is not a significant population of Purkinje cells that does not express detectable levels of Calbindin at older ages. Calbindin-labeled cells were easier to distinguish than those labeled with NeuroTrace. Taken together, Calbindin labeling appears to be a better means to detect more Purkinje cells than a Nissl stain like NeuroTrace.

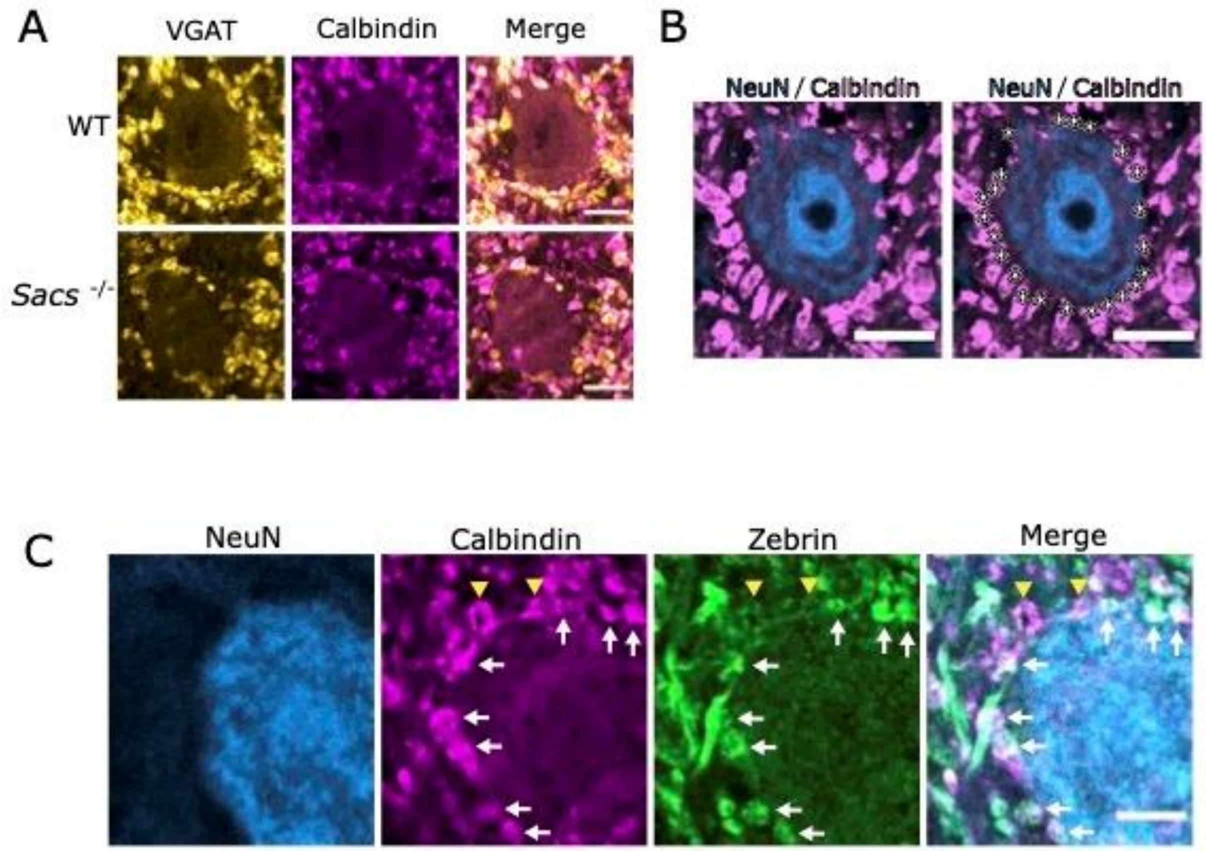

Supplementary Figure 2. **Calbindin-positive puncta on CN neurons likely reflect functional presynaptic synaptic terminals from Purkinje cells.**

(A) Representative images of calbindin-positive puncta onto large (>15 µm) CN neurons from the anterior interposed nucleus. Calbindin-positive puncta (purple, middle) are largely VGAT-positive (yellow, left, and right, merge), suggesting that they are functional presynaptic GABAergic terminals. Scale bar = 10 µm. **(B)** Example image (left) with detected puncta (right) shows the criteria for measuring puncta on CN neurons. **(C)** Individual CN neurons receive both zebrin-positive (green, middle, and white in merge, right) and zebrin-negative (purple in merged image, right) Purkinje cell puncta (purple Calbindin label, second panel).

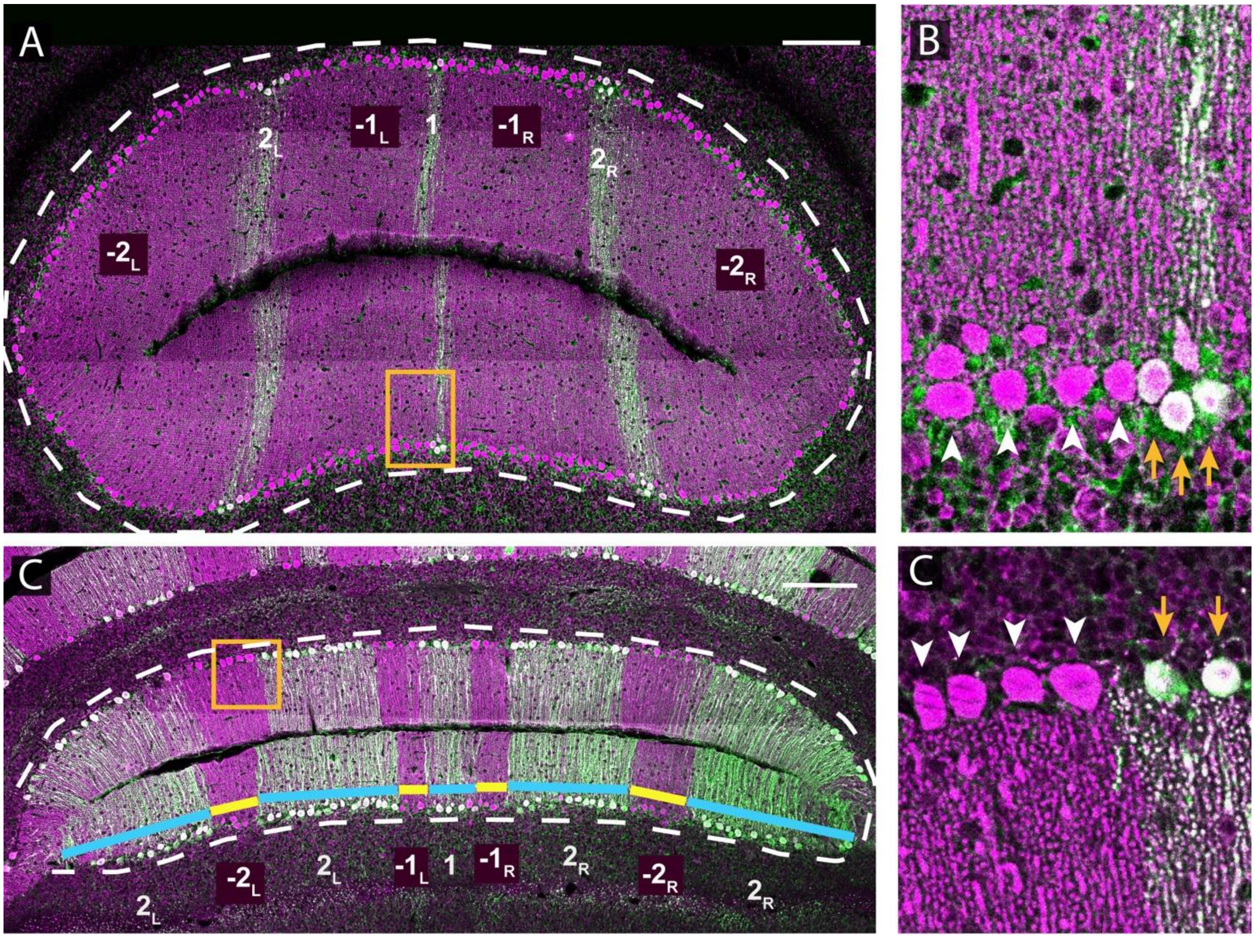

Supplementary Figure 3. **Example of analysis of Purkinje cell band and density measurements.**

(A) Sample image from an anterior lobule section. Zebrin-negative bands and cell bodies are shown in magenta (pseudo color), and zebrin-positive bands and cell bodies are shown in green (pseudo color). The dashed line denotes the area adjacent to where Purkinje cell layer and lobule length was measured (measurement was taken directly next to the Purkinje cell layer, but offset here for illustrative purposes). Purkinje cell counts were normalized to the length of the Purkinje cell layer. **(B)** Zoomed-in view of area within the orange box in **(A)**. White arrow heads denote zebrin-negative cells and yellow arrows denote the zebrin-positive cells. **(C)** Sample image from a posterior lobule section, colours and dashed line as above in **(A)**. Band widths were measured as indicated for zebrin-positive (blue) and zebrin-negative (yellow) lines superimposed on the ventral part of the lobule. **(D)** Zoomed-in view of area within the orange box in **(C)**. Arrow heads denote zebrin-negative cells and yellow arrows denote zebrin-positive cells. Scale bar= 200 μm.
